# 

**Published:** 1882-09

**Authors:** 


CONTENTS.

| | Page. | | Page. |
|---|---|---|---|
| Consumption | 435 | Frost Work | 452 |
| Climate for Consumptives | 441 | Sun Baths | 452 |
| The Nice Young Man | 442 | Mind and Charcoal | 454 |
| Too White Flour | 443 | Facts for the People | 459 |
| Retiring from Business | 443 | Care of the Eyes | 460 |
| Who are Happiest? | 447 | Sleeping | 461 |
| Apples | 450 | Sickness and Death | 462 |
| Thinking Brag | 451 | Bravery | 462 |

